# Protein S Deficiency and COVID-19: A Brutal Combination Leading to Acute Submassive Bilateral Pulmonary Embolism

**DOI:** 10.7759/cureus.41560

**Published:** 2023-07-08

**Authors:** Yashitha Chirumamilla, Yaman Almerstani, Huda Marcus, Ghassan Bachuwa

**Affiliations:** 1 Internal Medicine, Hurley Medical Center, Flint, USA; 2 Internal Medicine, Michigan State University College of Human Medicine, Flint, USA; 3 Internal Medicine, Hurley Medical Center, Michigan State University College of Human Medicine, Flint, USA

**Keywords:** mcconnell’s sign, antithrombotic therapy, submassive pulmonary embolism, covid-19 hypercoagulability, protein s deficiency

## Abstract

Protein S deficiency is a form of inherited thrombophilia that occurs due to low levels of or improper function of protein S. The role of protein S is to inactivate procoagulant factors, and a deficiency results in an increased risk of thrombotic events. The coronavirus disease 2019 (COVID-19) infection has also been studied to increase the risk of venous thromboembolism (VTE) due to an interplay of several mechanisms. However, the risk of VTE in patients affected by both of these disease processes simultaneously has not been thoroughly studied, and so recommendations regarding routine screening and prophylaxis of VTE have also not been established. We discuss the case of a 46-year-old woman with a past history of protein S deficiency and a recent COVID-19 infection who presented with complaints of shortness of breath. Upon examination, she was found to be hypoxic and tachycardic. A computed tomography angiography of the chest was done and revealed acute submassive bilateral pulmonary embolism with right heart strain and pulmonary infarcts. She was initially treated with intravenous heparin and later transitioned to oral anticoagulation for a minimum of six months.

## Introduction

Protein S deficiency is a relatively rare genetic disease resulting in low levels of or decreased activity of protein S. It can be diagnosed as early as infancy but is most commonly discovered in the third or fourth decade of life. Protein S plays a vital role in the coagulation system, and the lack of it predisposes individuals to develop thrombi. Patients affected by coronavirus disease 2019 (COVID-19) have been shown to have a higher risk of developing thrombi as well. The exact pathophysiology behind it is not fully clear, but several mechanisms have been identified. In this case, we will discuss the coexistence of these conditions and the increased risk they could pose for thrombus formation.

## Case presentation

A 46-year-old Caucasian woman presented to our facility with complaints of shortness of breath and hypoxia. She had a past medical history of protein S deficiency, diagnosed 18 years earlier after undergoing a miscarriage, as well as hypertension, asthma, chronic iron deficiency anemia, and gastroesophageal reflux disease (GERD). She had a mild variant of the COVID-19 infection three months earlier with no hospitalization or oxygen requirements. However, since then she has had several episodes of pneumonia and completed antibiotic courses and steroids but has not returned to baseline, so she visited her pulmonologist. At the office, she was saturating at 84% on room air and was immediately referred to the emergency department.

Upon arrival, the patient was tachycardic with a pulse of 125, tachypneic with a respiratory rate of 30, and saturating well on 2L of oxygen via nasal cannula. A physical examination was significant for mild respiratory distress. Laboratory evaluation was significant for leukocytosis with a white blood cell (WBC) count of 22k, normal hemoglobin and platelet counts, and negative troponins. Given her hypoxia and tachycardia, a computed tomography angiography (CTA) of the chest was performed and revealed acute bilateral pulmonary emboli involving the right pulmonary artery with extension into the right and left segmental and subsegmental arteries.

**Figure 1:**
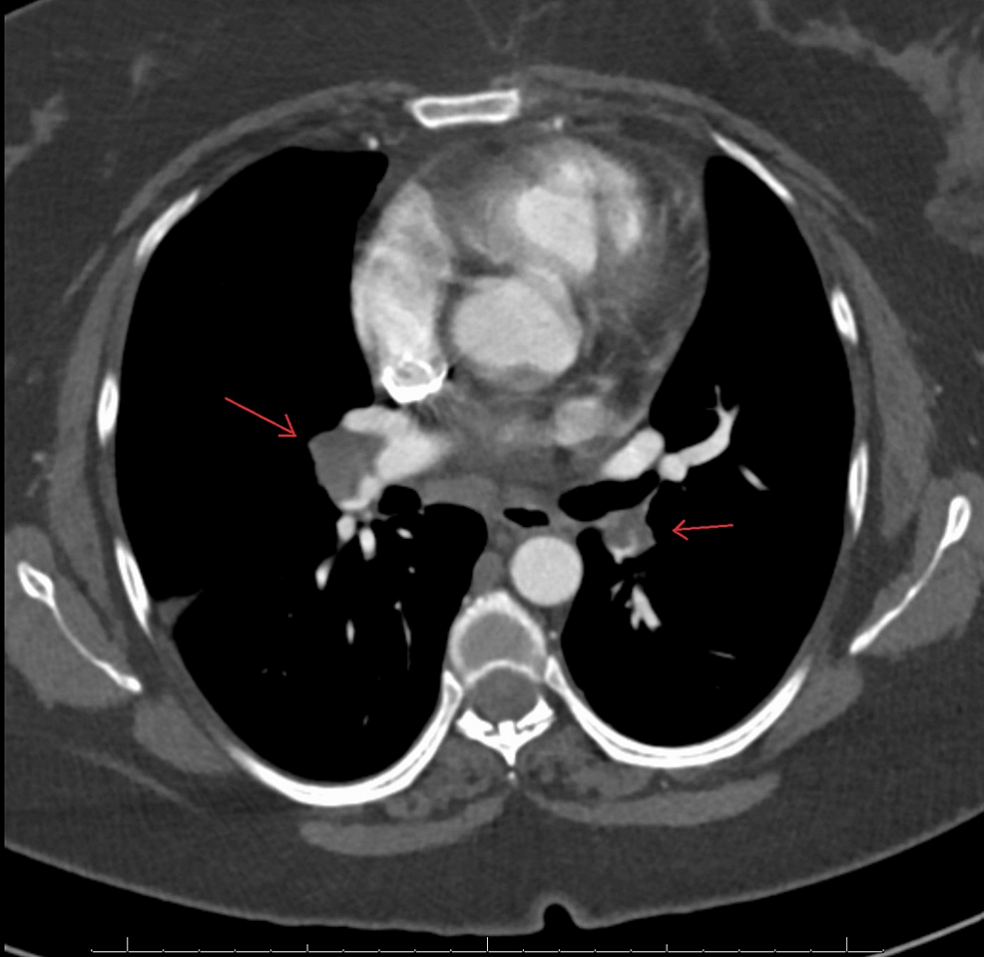
A CTA of the chest in axial view demonstrates acute bilateral pulmonary emboli involving the right pulmonary artery distally with extension into some of the right lower lobe and right middle lobe segmental and subsegmental arteries and some of the left lower lobe and left upper lobe segmental/subsegmental arteries and mildly enlarged pulmonary arteries. CTA: computed tomography angiography

Right heart strain was suspected with an enlarged right ventricle and a right ventricle to left ventricle (RV/LV) ratio of 1.6.

**Figure 2:**
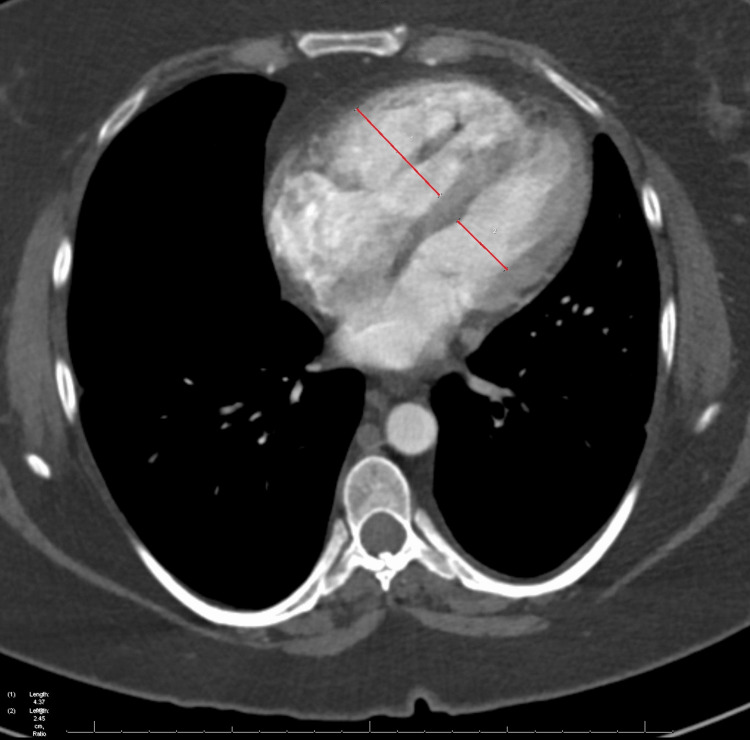
A CTA of the chest in axial view demonstrates right heart strain with an enlarged RV in comparison with the LV. The RV/LV ratio was measured to be 1.6. CTA: computed tomography angiography; RV: right ventricle; LV: left ventricle

Peripheral opacities were visualized in the right upper and left lower lobes of the lung, representing developing pulmonary infarcts. She was treated with intravenous heparin and underwent echocardiography, which demonstrated normal systolic function, a mildly dilated right atrium and ventricle, findings consistent with McConnell’s sign, an elevated right ventricular systolic pressure (RVSP) of 59mmHg, and a filling defect in the right ventricle most likely a mobile thrombus.

**Figure 3:**
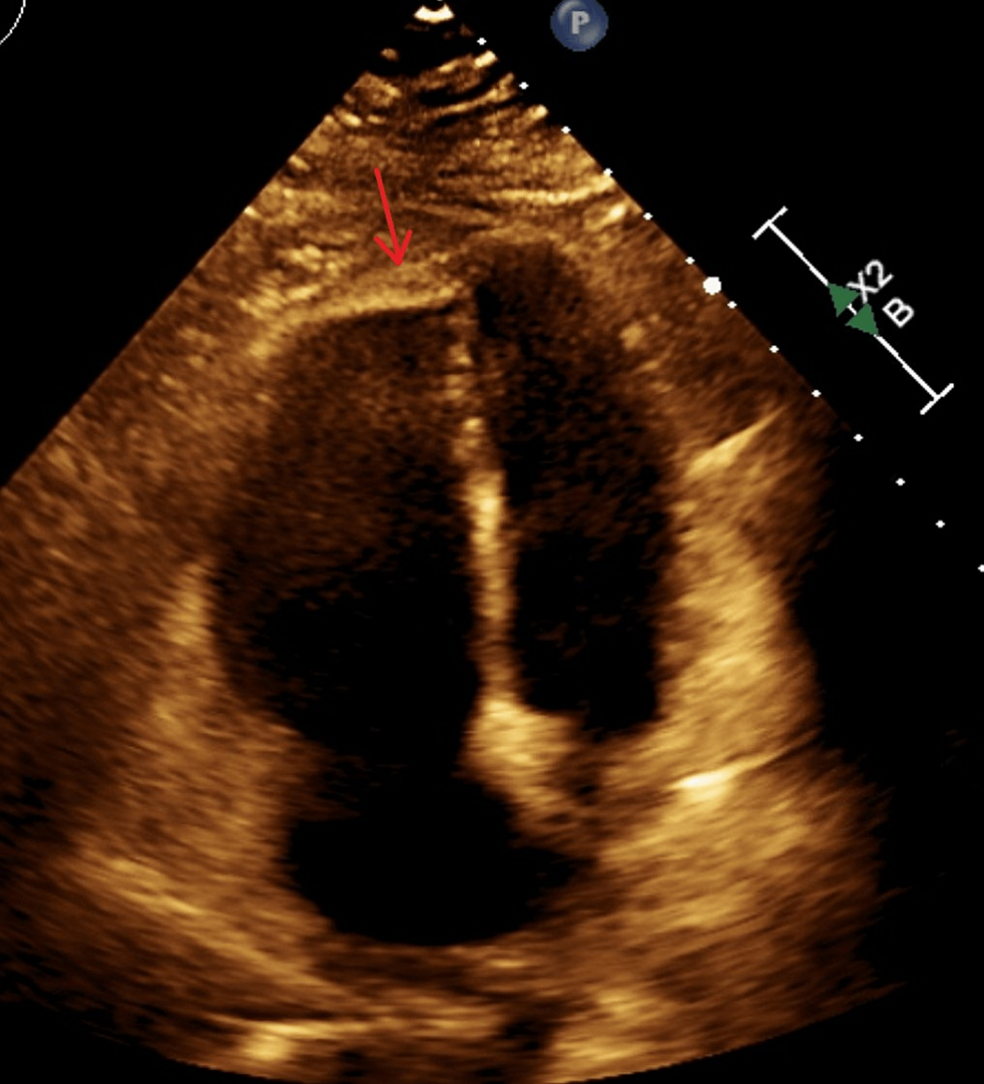
Echocardiography in a 4-chamber view shows a grossly dilated RV and a normal-sized LV. The arrow is depicting McConnell’s sign, in which the right ventricular apex moves inward with contraction, whereas the right ventricular free wall moves outward with contraction due to akinesis. RV: right ventricle; LV: left ventricle

Cardiology was consulted and discussed the risks and benefits of a thrombectomy with the patient, but she preferred medical management and was subsequently transitioned to a direct oral anticoagulant (DOAC). She was deemed fit for discharge with home oxygen. She was advised to follow up with her cardiologist within four weeks for a repeat echocardiography to evaluate her right ventricular thrombus and to continue her DOAC therapy for at least six months given her history of protein S deficiency.

## Discussion

Thrombophilia, or hypercoagulability, is associated with an increased risk of thrombus formation within blood vessels. It is caused by abnormalities in the coagulation cascade or the anticoagulation system. Protein S deficiency is an inherited thrombophilia caused by reduced activity or impaired function of protein S, a glycoprotein with anticoagulant properties. Patients with protein S deficiency have a high risk of recurrence of venous thromboembolism (VTE), with an annual incidence of recurrence after the first event of 8.4% [1]. Thrombophilia can also be acquired, as it is in the setting of COVID-19. The pathophysiology of hypercoagulability in COVID-19 is multifactorial and involves endothelial dysfunction, the release of pro-inflammatory cytokines, enhanced platelet activity, an imbalance of coagulation factors, and complement activation [2].

A study was conducted to review the impact of inherited thrombophilias in COVID-19; however, it was inconclusive in deeming there is an increased risk of VTE in those with thrombophilias. In fact, the study found that most patients with severe thrombophilia had fewer thrombotic events during a COVID-19 infection, likely because they were already taking anticoagulant medications [3]. Another prospective cohort study was performed to determine an increased risk of COVID-19-associated VTE in the presence of inherited thrombophilias. The study analyzed six different genetic polymorphisms that result in inherited thrombophilia, including a prothrombin mutation and a coagulation factor XI mutation. It was found that two of the six studied polymorphisms were associated with a higher risk of VTE [4]. Another case-control study measured the levels of protein C and protein S in those affected by COVID-19 in comparison to the general population. They concluded that protein S levels were lower in patients infected with COVID-19 and that the levels served as a marker for disease severity and mortality, which suggests that protein S plays a role in the thromboembolism associated with COVID-19 infections [5].

Currently, according to the COVID-19 Treatment Guidelines Panel, there is a recommendation against the use of anticoagulation for VTE prophylaxis in nonhospitalized COVID-19 patients. However, this does not apply to patients who have other indications for anticoagulation [6]. In our patient’s case, she did not meet the criteria for anticoagulation for COVID-19-associated VTE because she was never hospitalized. There is also insufficient data to state whether routine screening for VTE in all COVID-19 patients is beneficial, irrespective of their coagulation profile and clinical presentation [5].

Current recommendations for prophylactic anticoagulation in patients with protein S deficiency are dependent on previous personal or family history of VTE as well as additional risk factors. Our patient had no family history of VTE and had no previous thrombotic event, so anticoagulation was indicated for her only in high-risk scenarios such as pregnancy, surgery, prolonged immobilization, or acute medical illness requiring hospitalization [7].

The hypercoagulability of both protein S deficiency and COVID-19 ultimately made her prone to developing a bilateral pulmonary embolism with right heart strain, which poses the possibility of the conditions having a synergistic effect. Further research must be done to determine the thrombotic risk for patients with inherited thrombophilias and COVID-19 infection to better guide the optimal approach to anticoagulant regimens and possibly prevent such thrombotic events [3,4].

## Conclusions

Identifying risk factors, such as inherited thrombophilias that can predispose to COVID-19-associated VTE, is crucial to determining the overall risk of thrombosis in patients affected by the COVID-19 infection. As demonstrated by our patient, the severity of the infection may not be the best predictor of the risk for thrombosis, and the current recommendations only suggest anticoagulation for COVID-19-affected patients requiring hospitalization. Inherited thrombophilias and COVID-19 infection could have a synergistic effect in regards to hypercoagulability; however, larger-scale research will give us better insight into the interplay of this combination and help to optimize prophylactic anticoagulation guidelines.

## References

[1] Badulescu OV, Sirbu PD, Filip N (2022). Hereditary thrombophilia in the era of COVID-19. Healthcare (Basel).

[2] Abou-Ismail MY, Diamond A, Kapoor S, Arafah Y, Nayak L (2020). The hypercoagulable state in COVID-19: incidence, pathophysiology, and management. Thromb Res.

[3] de la Morena-Barrio ME, Bravo-Pérez C, de la Morena-Barrio B (2021). A pilot study on the impact of congenital thrombophilia in COVID-19. Eur J Clin Invest.

[4] Stevens H, Canovas R, Tran H, Peter K, McFadyen JD (2022). Inherited thrombophilias are associated with a higher risk of COVID-19-associated venous thromboembolism: a prospective population-based cohort study. Circulation.

[5] Elshafie A, Foda E, Yousef MM, El-Naby KA (2023). Evaluation of protein C and S levels in patients with COVID-19 infection and their relation to disease severity. Egypt J Intern Med.

[6] (2023). Antithrombotic Therapy in Patients With COVID-19. https://www.covid19treatmentguidelines.nih.gov/therapies/antithrombotic-therapy/..

[7] Bauer KA (2023). Protein S Deficiency. Uptodate.com.

